# Adherens Junction Distribution Mechanisms during Cell-Cell Contact Elongation in *Drosophila*

**DOI:** 10.1371/journal.pone.0079613

**Published:** 2013-11-04

**Authors:** Gabrielle Goldenberg, Tony J. C. Harris

**Affiliations:** Department of Cell & Systems Biology, University of Toronto, Toronto, Ontario, Canada; Instituto Gulbenkian de Ciência, Portugal

## Abstract

During *Drosophila* gastrulation, amnioserosa (AS) cells flatten and spread as an epithelial sheet. We used AS morphogenesis as a model to investigate how adherens junctions (AJs) distribute along elongating cell-cell contacts *in vivo*. As the contacts elongated, total AJ protein levels increased along their length. However, genetically blocking this AJ addition indicated that it was not essential for maintaining AJ continuity. Implicating other remodeling mechanisms, AJ photobleaching revealed non-directional lateral mobility of AJs along the elongating contacts, as well as local AJ removal from the membranes. Actin stabilization with jasplakinolide reduced AJ redistribution, and live imaging of myosin II along elongating contacts revealed fragmented, expanding and contracting actomyosin networks, suggesting a mechanism for lateral AJ mobility. Actin stabilization also increased total AJ levels, suggesting an inhibition of AJ removal. Implicating AJ removal by endocytosis, clathrin endocytic machinery accumulated at AJs. However, dynamin disruption had no apparent effect on AJs, suggesting the involvement of redundant or dynamin-independent mechanisms. Overall, we propose that new synthesis, lateral diffusion, and endocytosis play overlapping roles to populate elongating cell-cell contacts with evenly distributed AJs in this *in vivo* system.

## Introduction

Adherens junctions (AJs) mediate cell-cell adhesion essential for the structure and function of the epithelial sheets that coat our body compartments [1,2,3,4,5]. Classical cadherins (e.g. *Drosophila* E-cadherin; DE-cad) are transmembrane adhesion receptors at the core of AJs. They mediate homophilic cell-cell adhesion through their extracellular domains and link to adaptor proteins through their cytoplasmic tails. β-catenin (*Drosophila* Armadillo; Arm) binds the cytoplasmic tail of classical cadherins and additionally interacts with the actin-binding protein α-catenin to form cadherin-catenin complexes. Through direct and indirect links to actin, α-catenin mediates interactions between cadherin-catenin complexes and actomyosin networks to form AJs. At maturity, AJs are found around the lateral circumference of epithelial cells, often with apicolateral enrichment, and, together with their associated cytoskeletal networks, form massive protein interaction networks spanning the epithelial sheets that form our organs.

Dynamic AJ remodeling is important for tissues to reorganize during development, homeostasis and disease [6,7,8,9,10]. Together with their potential to form tissue-wide protein interaction networks, AJs are also dynamically regulated. This regulation controls the amount of AJ proteins incorporated into AJs, their lateral mobility, and their removal from AJs. From the plasticity of migrating clusters of cells [11] to the stable epithelium of the organ of Corti [12], the protein interaction networks formed by AJs can have a range of dynamic properties. However, we are just beginning to acquire an integrated view of these dynamics and how they are regulated.

Disease and cell culture models have identified several major mechanisms of AJ remodeling; the control of AJ protein levels, the lateral mobility of AJ proteins through the plasma membrane (PM) and the endocytosis and recycling of AJ proteins [13,14,15,16]. The importance of controlling AJ protein levels has become particularly clear in cancer models in which protein reductions can result from transcriptional or post-transcriptional changes and have been linked to tissue breakdown and metastasis [13]. Once at the PM, optical tracking and trapping experiments have shown that AJ proteins outside of contacts can move laterally but are restrained by cytoskeletal corrals or tethers [17,18]. Within cell-cell contacts, larger puncta of cadherin-catenin complexes have been observed to undergo actin-dependent flows [19]. Additionally, surface labeling experiments have shown that AJ proteins can be endocytosed from the PM and recycled back [20]. The relative contribution of lateral mobility and endocytosis in distributing AJs along contacts is controversial. Photobleaching and inhibitor studies have indicated that AJ endocytosis and recycling is the dominant mode of AJ repositioning in confluent cultured epithelia [21], whereas cadherin constructs with their endocytic motifs mutated or deleted have also been shown to assemble and disassemble cadherin-catenin clusters and undergo lateral movements along the PM [22,23]. Resolving the relative contributions of AJ distribution mechanisms across different tissues is important for understanding how various tissues behave.

*Drosophila* is an excellent model for examining the major mechanisms of AJ remodeling in a developmental system [7,8,10]. Illustrating the importance of AJ protein levels, zygotic mutants of *shotgun* (*shg*; the gene encoding DE-cad) have dwindling amounts of maternally supplied *shg* gene product which first become functionally limiting in morphogenetically active tissues [24,25]. Additionally, larger puncta of cadherin-catenin complexes have been observed to move laterally within initially developing cell-cell contacts [26], and at more mature contacts when their links to actomyosin networks are weakened experimentally [27]. Endocytosis and recycling contributes to AJ remodeling during cell intercalation [28,29], as well as to AJ homeostasis in less active tissues [30,31,32,33]. A comprehensive analysis of AJ dynamics across columnar *Drosophila* epithelia, revealed that compared with mature epithelia, AJ proteins in the early embryonic ectoderm were resupplied with greater new synthesis, underwent more lateral mobility, and displayed lower immobile fractions [34]. These studies show that the three well-documented AJ distribution mechanisms are active *in vivo*. Also, it is evident that each mechanism can contribute to AJ positioning in the same tissue [33,34], raising the question of how these mechanisms are integrated at individual cell-cell contacts.

*Drosophila* amnioserosa (AS) morphogenesis provides a model for studying how AJs are distributed as cell-cell contacts elongate. Cell-cell contact elongation provides a challenge to AJs. To remain continuous they must populate the newly forming contact between the PMs of neighbouring cells. In one model of cell contact elongation, *Drosophila* oocyte follicle cells, AJs do not spread evenly but become discontinuous with cell contact elongation [35]. As the *Drosophila* AS forms at gastrulation, initially columnar epithelial cells rotate their contents by 90° to become flat squamous epithelial cells. As this transition occurs, the cells retain cell-cell contacts with their original neighbours and their apicolaterally localized AJs evenly populate the rapidly expanding contacts [36]. Other than relying on an intact actin cytoskeleton [36], it is unclear how this even distribution of AJs is maintained as the contacts elongate. However, the three major mechanisms for AJ distribution have the potential to contribute: (1) Total embryo AJ protein levels are increasing at gastrulation [37]; (2) AS cell elongation is coupled with a loss of AJ non-muscle myosin II (myosin hereafter) [36,38], suggesting a weakening of actomyosin networks and a potential for greater AJ lateral mobility; and (3) AJ endocytosis occurs at shortening contacts between intercalating cells of the germband found just next to the AS [28]. Here, we provide evidence that new AJ addition, AJ lateral mobility and AJ removal function in concert for robust AJ regulation as AS contacts elongate.

## Methods

### *Drosophila* stocks and antibodies

The following stocks were used: arm^249^;arm-Arm-GFP [39], sqh-Clc-mCherry [28], sqh-Sqh-GFP [40], ubi-DE-cad-GFP [41], shg^R69^ [42], and shi^ts^ [43]. Flies with constitutively expressed histone-GFP (gift from Andrew Wilde, University of Toronto) and yellow white (yw) were used as WT controls.

The following primary antibodies were used: rabbit anti-α-adaptin (1:50) [44], mouse anti-Armadillo (1:100; Developmental Studies Hybridoma Bank #N27A1), rat anti-DE-cadherin (1:100 Developmental Studies Hybridoma Bank #DCAD2), and mouse anti-Sec5 [45].

### Embryo fixation and staining

Embryos were collected at 25°C, 2-4 h after egg laying during which time AS morphogenesis occurs. Embryos were washed three times with 0.1% Triton X-100, dechorionated with 50% bleach for 5 min and then washed three times with 0.1% Triton X-100 to remove the bleach. Embryos were then fixed by adding equal amounts of heptane and fixative solution (3.7% formaldehyde in PBS) for 20 min. The bottom formaldehyde layer was then removed, and an equal amount of methanol was added to the remaining heptane. The mixture was shaken for 30 sec to devitellinize the embryos. The embryos were then washed with methanol and rocked for 1.5 h in NGS block solution (PBS with 0.1% Triton (PBT), 1% normal goat serum (NGS), 1% NaN_3_). Primary antibodies diluted in NGS block solution were added to the embryos and incubated with rocking at 4°C overnight. Embryos were then washed with PBT for 10 min three times, and then with NGS block solution for 30 min. Embryos were then incubated with secondary antibodies (diluted in NGS block solution at 1:500) for 2 h. Secondary antibodies were goat anti-rabbit, mouse or rat conjugated to Alexa Fluor 488, 568 or 647 (Invitrogen), and were pre-absorbed with fixed yw embryos before use. After washing with PBT three times for 10 minutes, embryos were transferred onto a slide, excess liquid was absorbed using a Kimwipe, and a drop of Aqua polymount solution (Polysciences) was added before placing a glass coverslip on top of the embryos.

### Jasplakinolide treatment

Dechorionated embryos were washed three times with 0.1% Triton X-100 and then three times with 0.9% NaCl. Then, equal amounts of octane and 0.9% NaCl (containing either the drug or carrier control) were added to the embryos and incubated for 30 minutes. Specifically, 6μl of 2μg/μl of Jasplakinolide (EMD Millipore) in DMSO, or 6μl of DMSO alone, were added to 600μl of 0.9% NaCl. The bottom NaCl layer was then removed, followed by removal of the top octane layer. Embryo fixation and staining was then carried out as above.

### Heat treatment

Embryos were collected at RT, 2-4 h after egg laying. After dechorionation and washing, all RT 0.1% Triton X-100 solution was removed, and 1.4ml of 37°C 0.1% Triton X-100 was added to the embryos, and the tube was transferred into a standard heat block set to 37°C for 30 minutes. Embryos were then fixed with 37°C heptane and fix solution components (the stock formaldehyde solution was not preheated) and nutated for 20 minutes in a 29°C incubator. Fix solution was then removed, and the devitellinization and staining protocol was then carried out as above.

### Live imaging preparations

Embryos were collected as above and washed with 0.1% Triton X-100, dechorionated with 50% bleach, rinsed again with 0.1% Triton X-100 and placed on a petriPERM membrane dish (Sigma). Excess liquid was absorbed with Kimwipes, embryos were positioned on their dorsal side under a dissecting microscope and a drop of halocarbon oil (series 700; Halocarbon Products) was added before adding the coverslip.

### Imaging, FRAP and iFRAP

Live and fixed images were taken at RT with a Quorum spinning disk confocal microscope (Quorum Technologies) with Zeiss 40x (NA 1.3) and 63x (NA 1.4) objective lenses and a Hamamatsu EM CCD camera with Velocity software (Improvision). Movies and images were taken with Z-stacks of 0.3μm. 

Cells were photobleached for 3 sec with an argon laser at the junctional level. For FRAP experiments, one long rectangular box with a length of 48-53 µm and a width of 4-6 µm was drawn length-wise in the A-P direction, spanning 7-12 elongating AS cell-cell contacts and was photobleached. For iFRAP, two long parallel rectangular boxes each with a length of 39-51 µm and a width of 5-8 µm were drawn length-wise in the A-P direction with a separation of 2-4.5 µm and were photobleached, leaving a rectangle containing 7-12 unbleached AJ regions between them. Cells were imaged for 2-3 min before photobleaching and up to 12 min after photobleaching.

### Total AJ fluorescence and height measurements

To categorize the stages of AS morphogenesis, the apical circumferences of early (21.6±2.5μm) and mid (44.2±5.3μm) cells were measured using the ‘line’ tool in the Velocity software. Imaris software (Bitplane) was used to compare the total signal intensities of specific cell contacts of early and mid AS cells following 3D surface rendering. Surface formation and exclusion criteria were optimized to specifically capture AJs in the renderings and were kept constant across all embryos in each experiment. The ‘cutting’ tool was used to isolate individual elongating cell-cell contacts—the 3D renderings were cut just close to, but did not include, the tri-cellular junctions at each end of the contact. The ‘intensity value’ gave the total signal intensity in the contact volume, and measures of the ‘ellipsoid axis’ provided the contact volume’s height, width and length. For DE-cad-GFP live imaging, cells were followed throughout AS morphogenesis and the same contacts were quantified at early and mid stages. These embryo values were normalized by dividing each early stage value by itself, and dividing the mid stage values by the early stage value of the same embryo. For fixed yw embryos stained for Arm and DE-cad, populations of cell contacts from early and mid stages were compared. All values from early and mid stages were normalized by dividing them by the average of all early stage values. Imaging of each replicate was done on the same day with the same settings. 

### Local AJ fluorescence measurements

To quantify local fluorescence intensities within contacts, local mean grey values were measured with Image J (NIH) from single confocal sections. The ‘oval’ tool was used and measurements were taken in the centre of all elongating contacts. All values from the early and mid stages were normalized by dividing them by the average of all early stage values. Imaging of each replicate was done on the same day with the same settings. The Image J ‘RGB Profiler’ plugin was used to compare α-adaptin, Sec5 and DE-cad intensities along line scans.

### FRAP analyses

Mean grey values were taken as a measure of average fluorescence intensity within regions of interest using Image J. Using the ‘rectangle’ tool, squares were drawn in the middle of the photobleached area along elongating contacts and measured for mean grey value at each time point. The size of the square was kept constant for all time points. To correct for background, the average mean grey value of 3-4 squares of the same size, measured within the field of view, but outside of the embryo, was subtracted from the mean grey value of the photobleached area, at each time-point. To correct for general photobleaching and new synthesis, the change in mean grey value of a larger square containing 5-7 whole cells outside of the photobleached area, was subtracted from values from the FRAP region at each time-point. Final intensities of the photobleached area were then corrected to 1 by dividing each value by the value immediately before photobleaching. Mobile fractions and t½ values were calculated as described [46].

### iFRAP analyses

To monitor intensity changes, mean grey values were measured with Image J. Using the ‘rectangle’ tool, squares were drawn in the middle of the unbleached junctional area (which had been isolated by photobleaching the areas above and below) along elongating contacts and measured for mean grey value at each time point. To obtain the intensity of the junctions alone, mean grey values of cytoplasmic areas directly adjacent to the unbleached junctions were subtracted from the unbleached mean grey value at each time point. To determine the change in unbleached intensities over time, unbleached intensities were corrected to 1 by dividing each value by the value at the first time-point after bleaching. Corrections for general photobleaching and new synthesis were completed as above (FRAP analyses). 

To monitor length changes, the ‘line’ tool was used in the Velocity software to measure the change in unbleached junctional length, contact length and apical circumference in μm. Contact length measurements were taken from tri-cellular junction to tri-cellular junction at each time point. Unbleached line length measurements were taken until blending of the bleached and unbleached lines made the unbleached line indiscernible. 

For direct comparisons, all parameters for each iFRAP contact were determined over the same time period. The iFRAP correlation analyses were done by first plotting the unbleached line length, contact length, and unbleached line intensity versus time for each contact separately. If any parameter was not able to be quantified for at least 3 time-points, the contact was excluded. For the parameter versus time graphs, R^2^ values were determined. For comparing the rate of change of one parameter versus another, at least one of the two parameters had to have an R^2^ value greater than 0.5 in its comparison with time. All graphical analysis, slopes and R^2^ values were completed in Excel.

### Statistics

Statistical comparisons were completed using Student’s t-tests (two tailed, two samples, unequal variance) in Excel.

## Results

**Figure 1:**
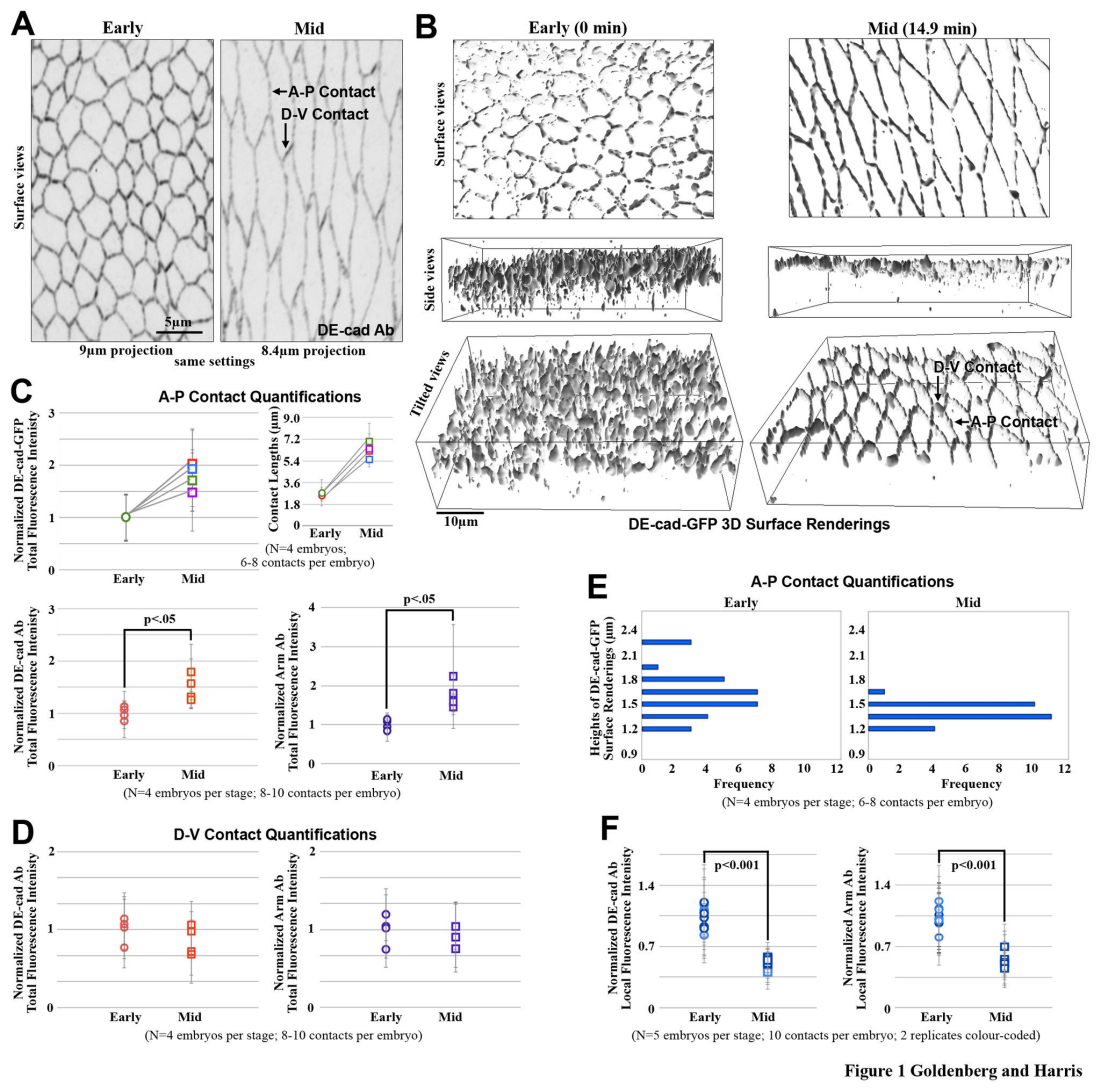
AJ changes along elongating contacts. (A) DE-cad staining during early and mid AS cell elongation. Elongating A-P and non-elongating D-V cell-cell contacts are indicated. (B) 3D surface renderings of live DE-cad-GFP at early and mid AS cell elongation shown with surface, side and tilted views. A-P and D-V cell-cell contacts are indicated. (C) Quantifications of the total fluorescence intensity levels of live DE-cad-GFP (top), alongside length changes of the corresponding contacts. Quantifications of the total fluorescent intensity levels of antibody-stained Arm and DE-cad along full A-P contacts at early and mid elongation (bottom). The live analyses followed and measured single AS contacts from early to mid cell elongation. The fixed analyses compared populations of AS contacts from early and mid cell elongation. (D) Quantifications of the total fluorescent intensity levels of antibody-stained Arm and DE-cad along full non-elongated D-V contacts at early versus mid cell elongation. (E) Histograms of live DE-cad-GFP surface rendering heights at elongating contacts for early and mid cell elongation. (F) Quantifications of local average fluorescence intensity levels of antibody-stained Arm and DE-cad at A-P contacts at early versus mid cell elongation.

To examine changes to AJs as AS cell-cell contacts elongate, we used antibodies or GFP-constructs for DE-cad or Arm to image core AJ proteins. ‘Early’ and ‘mid’ cell elongation were categorized by apical cell circumferences of 21.6±2.5μm and 44.2±5.3μm at AJs, respectively. Just after the onset of gastrulation, AS cells elongate along the dorsal-ventral axis of the embryo without cell intercalation [36]. From early to mid cell elongation, most cell contacts at the dorsal and ventral ends of AS cells (D-V contacts) do not change their length considerably, whereas cell contacts at the anterior and posterior sides of AS cells (A-P contacts) elongate by ~2-fold (Figure 1A-C). For this study, we focused on A-P contacts with this degree of elongation to probe the remodeling of AJs. Live imaging of DE-cad-GFP showed that this elongation occurs in 10.4±3.1 min (mean±SD; N=4 embryos).

### Total AJ protein levels increase along elongating AS contacts

Since total AJ protein levels begin increasing just prior to gastrulation [37], we hypothesized that new AJ synthesis and addition contributes to the AJs along elongating contacts. To determine if DE-cad was added to elongating contacts, we created 3D surface renderings of DE-cad-GFP at AJs (Figure 1B), and compared quantifications of total fluorescence intensity between early and mid stages within these selected volumes along elongating A-P contacts in live embryos. In these live embryos, individual A-P contacts were monitored between the two stages, and displayed a 1.8-fold increase in total DE-cad-GFP fluorescence intensity from early to mid cell elongation (Figure 1C). To assess the change in total AJ protein amounts with a separate approach, WT embryos were fixed and stained with DE-cad and Arm antibodies, and populations of A-P contacts were compared at each stage following 3D surface rendering. Between early and mid elongation, significant increases in fluorescence intensity occurred for DE-cad (1.5-fold) and Arm (1.7-fold) (Figure 1C). These results show that there is substantial new addition of AJ proteins at A-P contacts during the time that these contacts elongate during AS morphogenesis.

To determine if this new addition was specific to elongating contacts, the total fluorescence intensity quantifications were repeated for non-elongating D-V contacts in WT embryos stained with DE-cad and Arm. At these contacts there was no significant change in DE-cad or Arm intensity levels between early and mid cell elongation (Figure 1D). Thus, new addition of DE-cad and Arm appears to contribute mainly to populating AJs along elongating A-P contacts.

### The normal increase of AJ proteins along A-P contacts is not essential for AJ continuity as the contacts elongate

**Figure 2:**
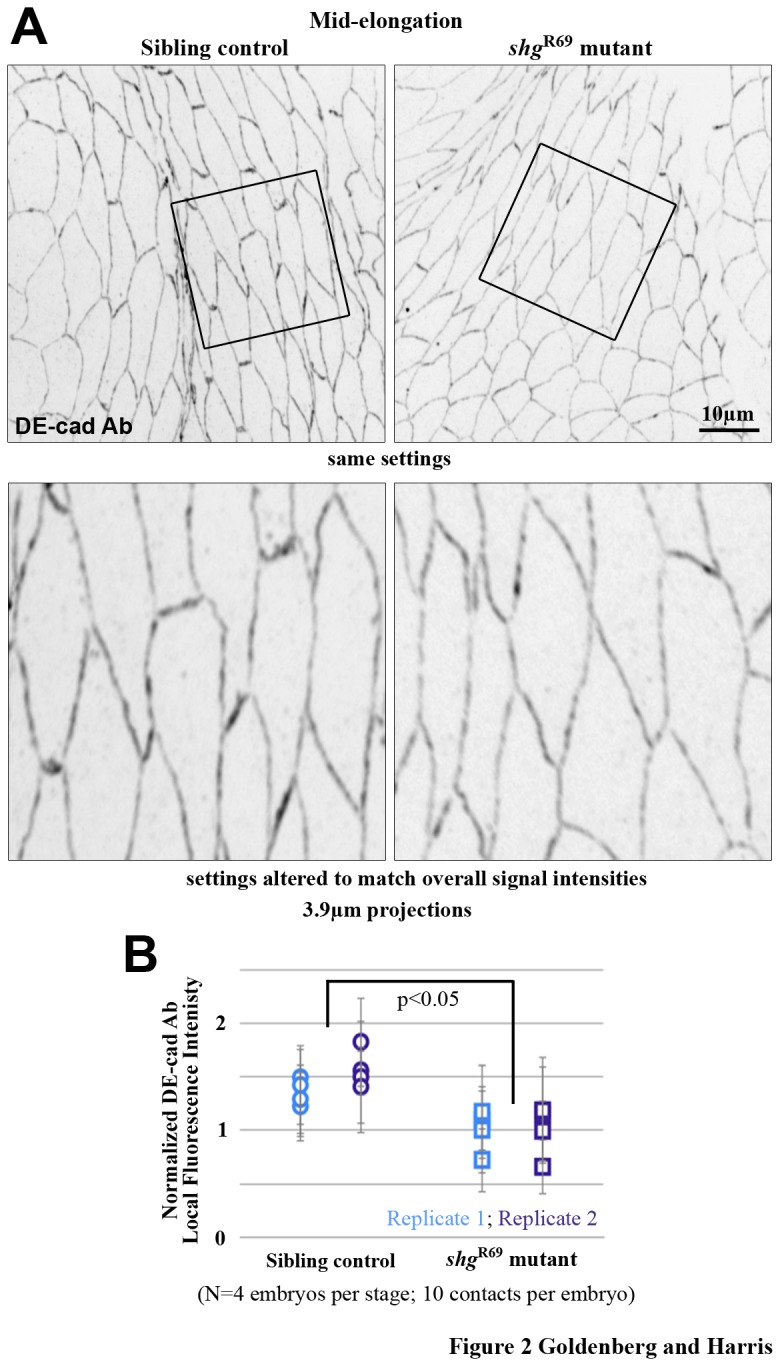
Increasing the total amount of AJ protein along elongating contacts is not essential for AJ continuity. (A) Fixed double balancer sibling control and shg^R69^ zygotic mutant AS cells at mid-cell elongation, stained with DE-cad antibodies. Magnified views (bottom) show no evidence of DE-cad fragmentation. (B) Local average DE-cad intensity measurements show a significant reduction at elongating contacts of shg^R69^ zygotic mutants compared to controls.

To determine the importance of the elevated DE-cad levels for proper AJ repositioning during AS morphogenesis, shg^R69^ zygotic mutant embryos (*shg* encodes DE-cad) were stained for DE-cad. Homozygous shg^R69^ embryos and homozygous GFP-balancer chromosome sibling controls were co-stained, co-mounted and co-imaged. At mid elongation, the total DE-cad intensity along A-P contacts was decreased by 1.4-fold in shg^R69^ mutants compared to the internal controls (Figure 2A-B). Thus, almost all of the normal increase of DE-cad was eliminated in this context (compared with Figure 1C). However, DE-cad staining at elongating contacts revealed no evidence of AJ fragmentation in the shg^R69^ mutants (Figure 2A; observed in 14 embryos). This even distribution of AJs along elongating A-P contacts with substantially reduced AJ proteins suggests that the normal increase in AJ proteins is largely not essential for maintaining AJ continuity, and that mechanisms for redistributing AJ proteins may be involved.

### AJ proteins appear to redistribute along elongating contacts

To test for redistribution of AJ proteins, we probed for changes in local AJ protein densities by determining average fluorescence intensity values in small regions along A-P contacts at early and mid cell elongation. Local intensities of DE-cad and Arm antibody staining measured in identically sized sub-regions of A-P contacts at early and mid elongation revealed average intensity decreases of ~50% (Figure 1F). Thus, local AJ protein levels decrease along A-P contacts as cells elongate. This decrease occurs in concert with the overall increase in levels along the full length of the contacts, suggesting that redistribution of AJ proteins occurs.

To determine if there was AJ reorganization in the apical-basal axis, we analyzed the major axes of live DE-cad-GFP 3D surface renderings. Visual inspection (Figure 1B) and quantification of the heights of the 3D renderings (Figure 1E) revealed a height decrease at A-P contacts from early to mid elongation. This change suggests that there is repositioning of AJs along the apical-basal axis of elongating A-P contacts during AS morphogenesis. In contrast, visual examination of non-elongated D-V contacts indicated that they often do not change their height from early to mid cell elongation (Figure 1B, arrows), implicating specific remodeling events along the elongating A-P contacts.

### Local AJ dynamics vary along A-P contacts at mid cell elongation

**Figure 3:**
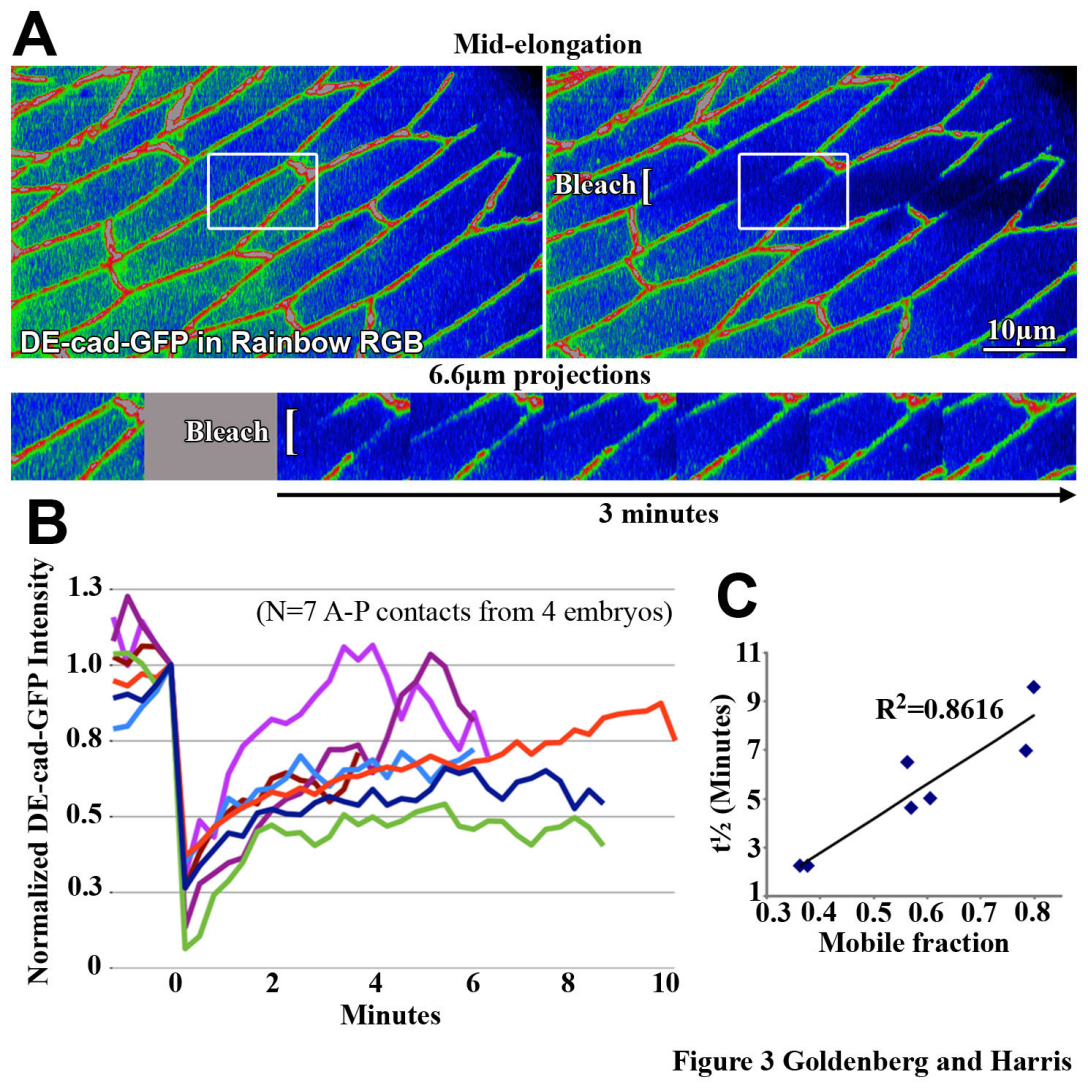
Local AJ dynamics vary along elongating contacts. (A) Mid stage AS cells before . (left) and after (right) photobleaching of DE-cad-GFP at elongating contacts. Square brackets indicate bleached region. Kymograph of white boxed region shows recovery over time. (B) Recovery curves of FRAP analyses indicate variable DE-cad-GFP recoveries. Data were normalized to the intensity level immediately prior to bleaching. (C) Plot of t½ values versus mobile fraction values for the recovery curves shown in B.

To initially assess the dynamics of AJ proteins along A-P contacts, FRAP analyses were performed in which local areas of DE-cad-GFP were photobleached at mid cell elongation (Figure 3A). Visual inspection of the recovery curves revealed highly variable behaviours (Figure 3B). Plotting t½ values versus mobile fraction values further illustrated the wide range of behaviours and showed that higher mobile fractions correlated with higher t½ values (Figure 3C). These variable recovery curves suggested that multiple and spatially distinct mechanisms might function to distribute AJs during contact elongation.

### AJ proteins laterally spread or condense along A-P contacts at mid cell elongation

**Figure 4:**
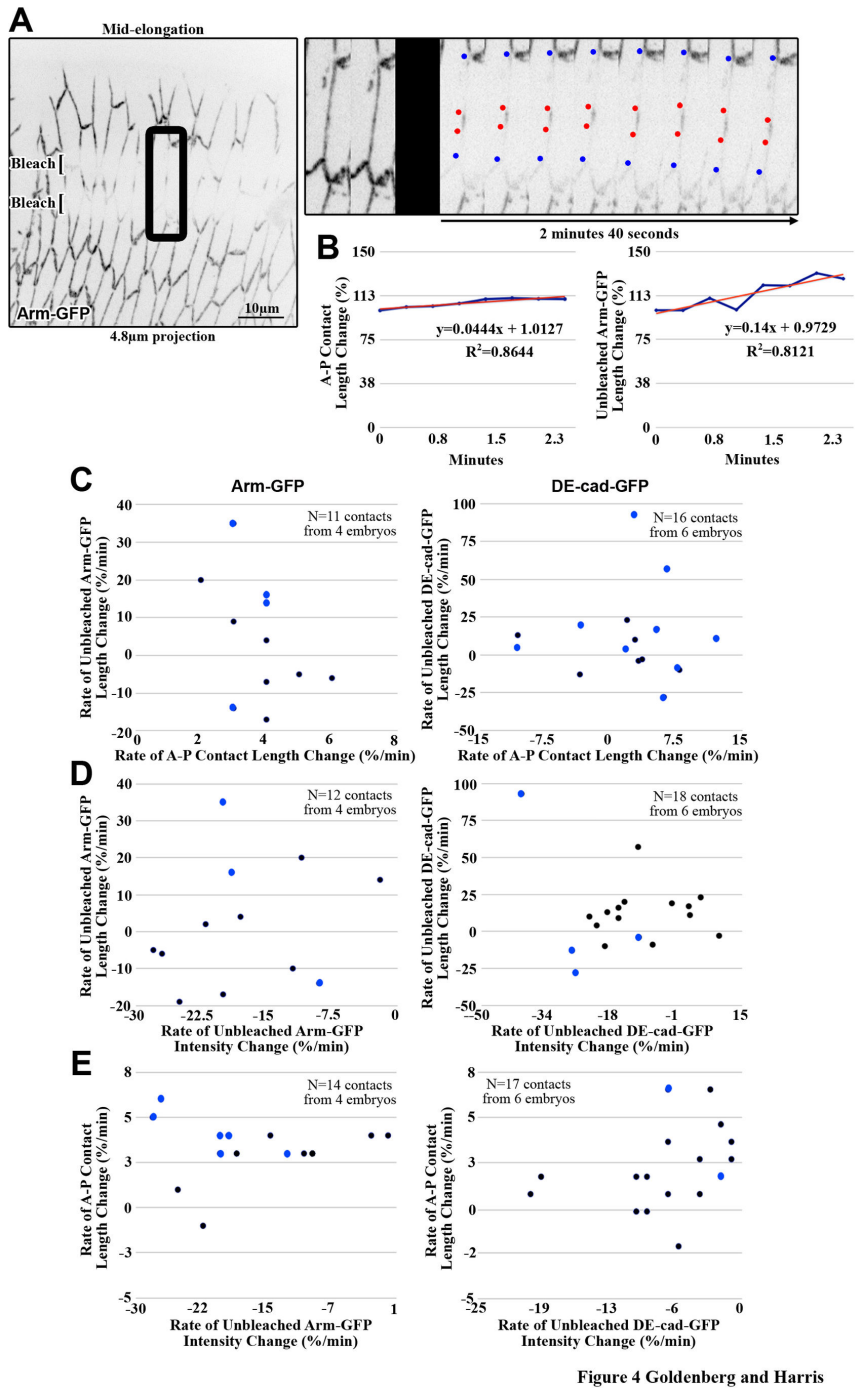
AJs exhibit lateral diffusion and internalization independent of contact elongation. (A) iFRAP of mid stage AS cells expressing Arm-GFP. Square brackets indicate bleached regions. Kymograph of black boxed region shows behavior of unbleached line over time. Blue dots track contact length change. Red dots track unbleached line length change. (B) Measurements of the above kymograph are plotted to determine slope and correlation coefficient (R^2^ value). (C-E) The slopes of graphs comparable to those in (B) are plotted if one had R^2^>0.5 (dark blue dots) or if both had R^2^ >0.5 (light blue dots) (see methods). (C) Plotting unbleached line length change versus contact length change for single elongating contacts shows no overall correlation between AJ lateral diffusion and contact elongation. (D) Plotting unbleached line length change versus unbleached intensity change for single elongating contacts shows no overall correlation between AJ lateral diffusion and AJ internalization. (E) Plotting contact length change versus unbleached intensity change for single elongating contacts shows no overall correlation between contact elongation and AJ internalization.

To isolate and monitor smaller groups of AJs we employed inverse FRAP (iFRAP) in which areas neighbouring the region of interest were photobleached (Figure 4A). Specifically, isolated non-bleached regions of DE-cad-GFP or Arm-GFP were analyzed to compare A-P contact elongation, AJ lateral mobility and AJ removal. Changes in total contact length, unbleached AJ region length and unbleached AJ region intensity were measured for the same A-P contacts over the same short time periods (from bleaching until the unbleached AJ region could no longer be distinguished).

To investigate lateral AJ movements, we measured changes in unbleached AJ region lengths. Both Arm-GFP and DE-cad-GFP regions showed fluctuations over time (Figure 4A), suggesting lateral AJ mobility. To assess how this lateral AJ mobility correlates with cell-cell contact elongation, we compared contact length changes to unbleached AJ region length changes for each contact separately (Figure 4B-C). We only included data in which at least one of the variables showed persistent and similar change over the time period (Figure 4B; see methods). With a positive correlation, we expected unbleached AJ regions to lengthen at the same rate as the contacts. However, there was no overall correlation between the contact length changes and unbleached AJ region length changes for either DE-cad-GFP (R^2^=0.002) or Arm-GFP (R^2^=0.205) (Figure 4C), suggesting that AJs flow laterally with independence from A-P contact elongation. Moreover, the local regions of DE-cad-GFP or Arm-GFP either increased or decreased their lengths regardless of the overall lengthening of the contacts. Thus, local mechanisms appear to move patches of AJs back and forth as the contacts elongate.

### Actomyosin networks along elongating contacts appear to affect AJ redistribution

**Figure 5:**
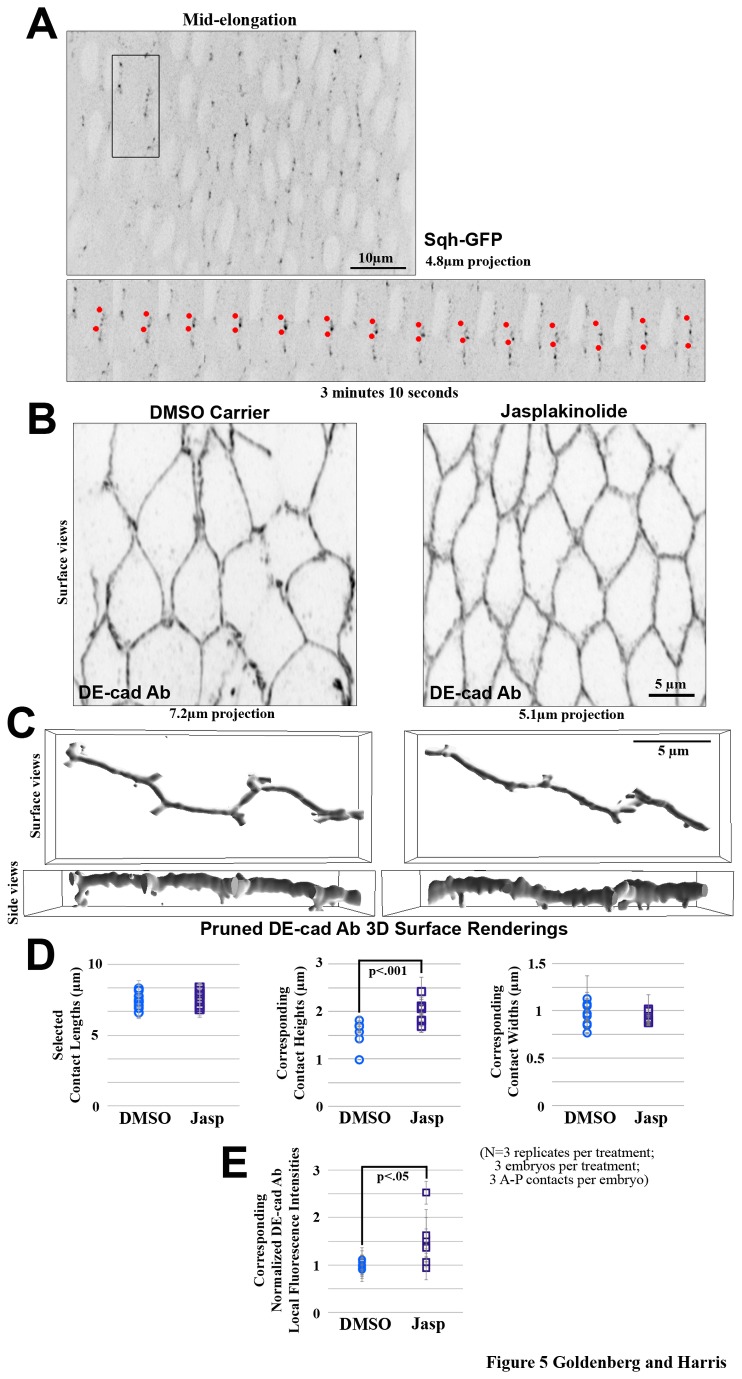
Myosin foci expand and coalesce along elongating contacts and actin stabilization reduces AJ redistribution. (A) Mid-elongated AS cells expressing Sqh-GFP. Kymograph of black boxed region shows behavior of myosin foci over time (red dots). (B) Mid-elongated AS cells treated with DMSO carrier control and jasp stained with DE-cad. (C) 3D surface renderings of AS A-P contacts at mid stage elongation showing apparent AJ lengths and widths (surface views) and heights (side views). Surrounding contacts were pruned to allow easier visualization. (D) Quantifications of AS contacts selected with the same lengths (left) showed an increase in DE-cad 3D surface rendering height but no change in width in jasp treated embryos versus DMSO carrier controls. (E) Quantifications of local average DE-cad fluorescence intensity showed an increase with jasp treatment versus DMSO carrier controls.

The actin cytoskeleton has been shown to play a role in corralling and tethering AJ molecules [17]. Interestingly, myosin is gradually lost from AS cell-cell contacts as they elongate [36,38]. To determine how myosin networks behave as they are lost, we live imaged Spagetti-squash-GFP (Sqh-GFP; *Drosophila* regulatory myosin light chain). Notably, fragmented patches of Sqh-GFP separately expanded and contracted along the elongating contacts (Figure 5A; observed in 6 embryos). This behaviour suggested that actomyosin networks may be locally influencing the mobility of AJs at elongating contacts.

To test whether the normal weakening of the actomyosin cytoskeleton during AS morphogenesis contributes to AJ redistribution, embryos were treated with jasplakinolide (jasp), a drug that stabilizes the actin cytoskeleton [47]. Jasp-treated embryos showed resistance to AS cell shape changes seen in DMSO control embryos (Figure 5B), suggesting that the jasp treatment rigidified the cortex of AS cells, as expected for actin stabilization. In the AS at mid cell elongation, the jasp treatment also resulted in more even and elevated DE-cad staining around the cells, compared to control (Figure 5B). In projections, the DE-cad-stained AJs also appeared thicker (Figure 5B). To visualize the thickening more clearly, 3D surface renderings were generated and single cell-cell contacts were examined after pruning away the surface renderings of surrounding contacts. These manipulations revealed an increase of surface rendering height without an increase to width (Figure 5C). To quantify these dimensional changes, elongating contacts within a specific length range were selected from the 3D surface renderings (Figure 5D). These surface renderings had increased heights with jasp treatment versus control, without changes in length or width (Figure 5D). To quantify the apparent increase in local DE-cad staining intensity, local intensities were compared between the sets of equal length contacts, and higher local DE-cad intensities were found with the jasp treatment versus control (Figure 5E). These data suggest that actin cytoskeleton turnover is important for permitting AJ re-distribution and removal from elongating AS cell contacts. It is important to note that the treatment alone affected AS cell shape and cell-cell interactions (as seen with the DMSO carrier control: Figure 5B), and thus the effects of actin on normal and artificially induced AJ remodeling are difficult to separate from these experiments alone.

### AJ proteins are removed along A-P contacts at mid cell elongation

Since the effects of actin stabilization suggested AJ proteins are normally removed from elongating contacts, we probed for this removal in our iFRAP data. Measurements of the change of local intensity within the isolated unbleached AJ regions revealed decreases over time independent of general photobleaching (Figure 4D; see methods for the photobleaching correction procedure), indicating that AJs are being locally redistributed or removed during AS morphogenesis. To determine if this reduction of local AJ intensity is in direct response to AJ lateral diffusion, we compared changes in local intensities to changes in unbleached AJ region lengths over the same time periods at the same contacts. As above, we only included data in which at least one of the variables showed persistent and similar change over the time period (see methods). No correlation was observed for either DE-cad-GFP or Arm-GFP (Figure 4D), indicating an independent removal mechanism. To test if this additional mechanism was induced in response to cell contact length change, changes in local intensities were also compared to changes in contact lengths for the same contacts but no correlation was observed (Figure 4E). Overall, these data suggest that AJs are removed along A-P contacts with independence from AJ lateral diffusion and A-P contact elongation.

### Endocytic and exocytic machinery localizes to AJs during cell elongation

**Figure 6:**
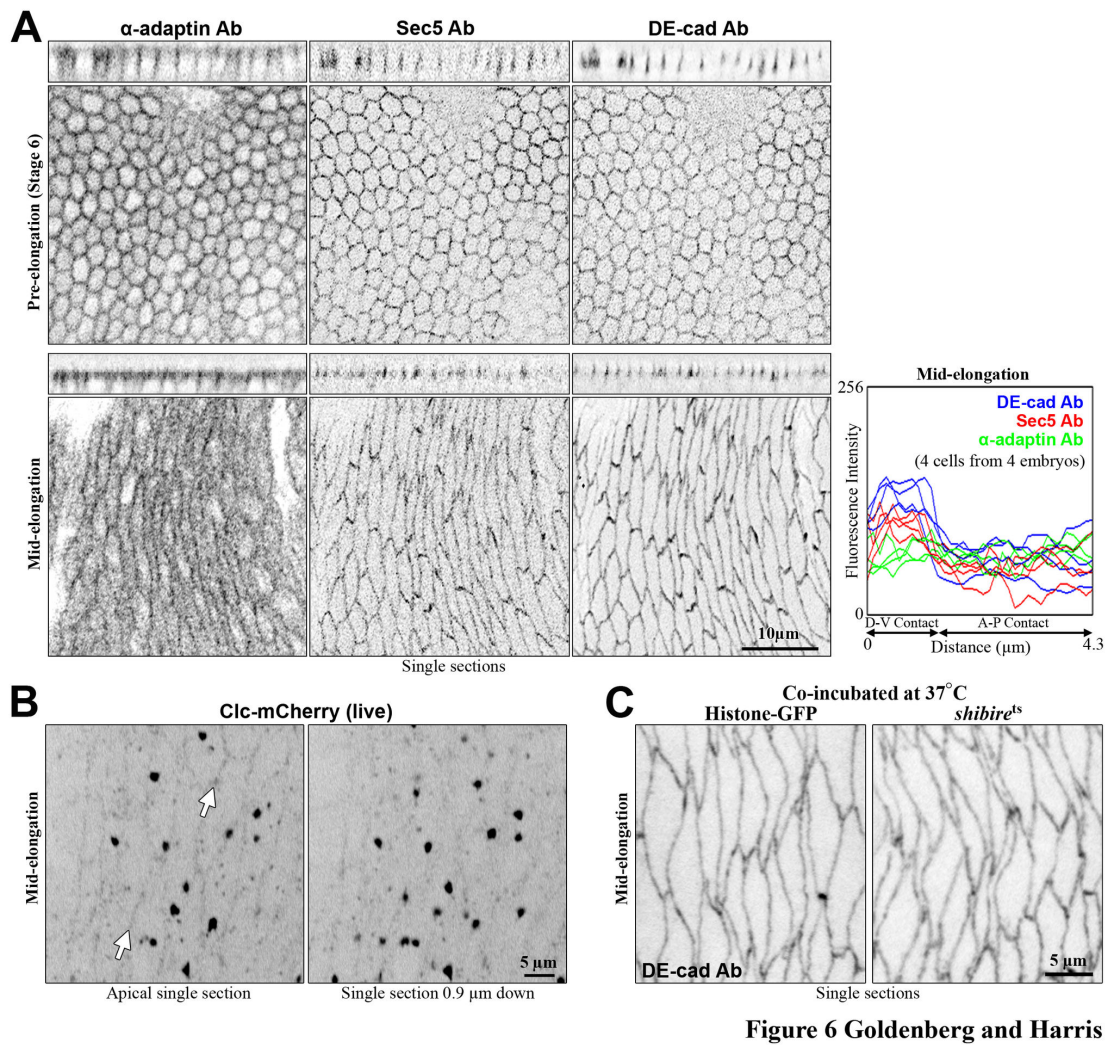
Endocytic machinery localizes at AJs along elongating contacts, but AJ redistribution occurs with dynamin disruption. (A) Prior to cell elongation, AS cells show Sec5 (an exocytic marker) and α-adaptin (an endocytic marker) at AJs but with uniform A-P and D-V localizations. At mid elongation, AS cells continue to have an even localization of α-adaptin at both D-V and A-P contacts, in contrast to Sec5 which is enriched at D-V contacts, similar to DE-cad (quantified at right with line scans starting along a D-V contact and then ending along a connected A-P contact). Each protein is stained with antibodies. (B) Mid-elongation AS cells expressing clc-mCherry shown at the AJ level and just below emphasizes enrichment at the AJ level (arrows). Multiple small clc-mCherry puncta are seen in the cytoplasm next to fewer large puncta. (C) Fixed shi^ts^ mutants stained with DE-cad after exposure to non-permissive temperature show no difference in AJ localization at mid elongation compared with co-treated and co-stained histone-GFP controls.

To test if endocytic machinery might be responsible for the removal mechanism, both endocytic (α-adaptin, a clathrin adaptor protein, and Clathrin light chain (Clc)) and exocytic (Sec5, a component of the exocyst complex) markers were imaged. Before and at mid-elongation, α-adaptin and Sec5 antibody staining and Clc-mCherry live imaging revealed apical enrichment in proximity to AJs (Figure 6A-B). Small and large Clc-mcherry puncta were also seen in the cytoplasm (Figure 6B). At mid cell elongation, all of the proteins were localized along the elongating cell-cell contacts (Figure 6A-B). However, Sec5 exhibited enhanced localization along non-elongating contacts, matching the planar polarity of DE-cad (Figure 6A). In contrast, α-adaptin staining was more even around the cell circumference and lacked enhanced localization at non-elongating contacts (Figure 6A), suggesting a different balance of exocytosis and endocytosis at the two contact types. Clc-mcherry was also more even and lacked the enhanced localization at non-elongating cell borders (Figure 6B), indicating a shared localization among clathrin-associated proteins. Overall, the presence of both endocytic and exocytic proteins at AJs along A-P contacts suggests that membrane recycling may contribute to the AJ remodeling during cell elongation.

### AJ remodeling at A-P contacts can occur with dynamin disruption during cell elongation

To test the role of endocytosis in AJ redistribution, temperature sensitive mutants of the gene encoding dynamin, shi^ts^ mutants, were examined at their non-permissive temperature. Using this approach to study the neighbouring ectoderm has revealed that dynamin only modestly affects AJs at intact cell-cell contacts [34], but that it is needed for AJ redistribution as cell-cell contacts are lost during cell intercalation [28]. As a positive control for our experiments, we exposed adult shi^ts^ flies to 37°C and they became temporarily paralyzed within minutes, consistent with the role of dynamin-dependent endocytosis in neurons [48]. We devised a protocol that immediately shifted early embryos to 37°C, maintained them at this temperature for 30 min to ensure that any embryos transitioning from early to mid amnioserosa cell elongation did so fully at the non-permissive temperature, and initiated embryo fixation at 37°C to avoid a reversal of the dynamin disruption (see methods). However, incubating shi^ts^ embryos at 37°C with this protocol produced no apparent changes to DE-cad antibody staining fluorescence or localization pattern, compared to co-incubated and co-stained histone-GFP controls (Figure 6C). This lack of effect suggests that AJs can evenly populate the elongating A-P contacts independently of full dynamin activity, possibly through low dynamin activity, redundant distribution mechanisms, dynamin-independent endocytosis, or a combination of these effects.

## Discussion

We have documented three major behaviours of AJ proteins as AS cell-cell contacts elongate: new addition to the contacts, lateral movement along the contacts, and removal from the contacts. Once delivered to the contacts, AJ proteins appear to be redistributed by the expansion and contraction of fragmented actomyosin networks along the contacts, and by endocytosis from the contacts. However, the even distribution of AJ proteins could not be perturbed by singly disrupting any of these mechanisms, suggesting that they function redundantly for robust localization of AJs during AS morphogenesis.

We quantified a ~50% increase in total AJ proteins along elongating contacts from early to mid cell elongation. The exocyst complex closely colocalizes with AJs at all cell-cell contacts in the tissue, suggesting a role in DE-cad exocytosis, as evident in other *Drosophila* tissues [32]. However, eliminating this net new addition has no noticeable effect on the even distribution of DE-cad along elongating contacts. Notably, within 2h of gastrulation, these mutants begin the lose cell-cell adhesion in the ventral neurectoderm, as holes left from delaminating neuroblasts fail to reform [25]. At this later stage, cell-cell adhesion is still maintained in the AS in the mutants, and the residual maternally supplied DE-cad remains remarkably continuous along the fully elongated contacts (data not shown). Thus, despite the rapidly expanding circumferences of the cells, net new addition of AJ proteins is not essential, and a stable pool of AJ proteins can apparently be effectively redistributed to maintain AJ continuity and cell-cell adhesion.

The fact that total AJ protein levels increase by only ~1.5-fold while the elongating cell-cell contact lengths increase by ~2-fold, suggests that local AJ densities decrease, and thus that AJ proteins are locally lost either though lateral movement or removal from the membrane. Indeed, direct measurements of AJ proteins showed a ~50% decrease in local intensities, and the apparent heights of AJs decreased as well. Furthermore, iFRAP analyses revealed that local AJ patch lengths often increase, or decrease, at rates of 25%/min. Additionally, the local intensities of these patches can drop by up to 30%/min. By quantifying multiple parameters for the same AJ region over time, we found that decreasing AJ protein levels did not correlate with the lateral spreading of the complexes, suggesting removal from the membrane. Notably, neither the lateral AJ movements nor the AJ losses correlated directly with the lengthening of the overall contacts, suggesting that the redistribution mechanisms are not directly triggered by contact elongation. Instead, we propose that local mechanisms result in constitutive expansions and condensations of cadherin-catenin clusters along the contacts, as well as constitutive removal, recycling and re-addition of AJ proteins along the contacts. These mechanisms would result in AJ plasticity that could allow pre-existing AJ proteins to populate elongating cell-cell contacts as they form.

A key element regulating AS cell shape change appears to be the loss of actomyosin networks from cell-cell contacts [36,38]. It is evident that these actomyosin networks restrain protrusive microtubule bundles from initiating elongation of the apical domain and cell rotation [36]. Thus, their weakening appears to permit the cell shape change. Here, we find that experimentally stabilizing actin networks rigidifies the cell cortex and also inhibits AJ redistribution. Thus, the normal weakening of actomyosin networks may contribute to both AS cell shape change and AJ plasticity along elongating contacts.

Weakening actomyosin networks could promote AJ remodeling in two ways: by increasing lateral mobility or by increasing endocytosis. Actin is known to tether and restrain AJs in many systems [1,3,4]. For example, in the ectoderm neighbouring the AS, actomyosin levels at AJs are normally maintained at higher levels than in the AS [38], but disrupting actin or α-catenin leads to the lateral mobility of AJ puncta with residual patches of actin attached [27]. With the normal loss of myosin from AS cells, we find that its networks fragment, and that these fragments expand and contract. We propose that these networks may expand and condense associated cadherin-catenin clusters. Also, the loss of actomyosin networks from the AS may contribute to AJ endocytosis, consistent with the observed increase in AJ protein levels at contacts with actin stabilization.

As discussed in the Introduction, there are clear examples where single AJ remodeling mechanisms play a dominant role in controlling cell-cell adhesion. For example, perturbing dynamin activity has a dramatic effect on AJ localization in the pupal notum [30,31], but has no clear effect at elongating AS cell-cell contacts. Such differences may be due to the relative effectiveness of other AJ distribution mechanisms in the tissues. For example, new synthesis of AJ proteins is much lower in the pupal notum versus the early embryo [34]. Tissues with multiple effective AJ distribution mechanisms may maintain adhesion more robustly. Defining such redundancies and their contributions to robust cell-cell adhesion is important for understanding different developmental and homeostatic processes, as well as different disease states, e.g. the propensity of cancerous tissues to metastasize.
